# P-922. Intracranial Pressure Phenotypes and Mortality Among People Treated for HIV-Related Cryptococcal Meningitis in Uganda

**DOI:** 10.1093/ofid/ofae631.1113

**Published:** 2025-01-29

**Authors:** Elizabeth L Schwartz, Minda Liu, Enock Kagimu, Edwin Nuwagira, Lillian Tugume, Kenneth Ssebambulidde, Isaac Turyasingura, Derrick Kasozi, Enos Kigozi, Samuel Okurut, Abdu kisekka Musubire, Caleb Skipper, Mahsa Abassi, Ann Fieberg, Conrad Muzoora, David Meya, David R Boulware

**Affiliations:** University of Minnesota, Minneapolis, Minnesota; University of Minnesota, Minneapolis, Minnesota; Infectious Diseases Institute, Makerere University, Kampala, Kampala, Uganda; Mbarara University of Science and Technology, Mbarara city, Mbarara, Uganda; Infectious Diseases Institute, Makerere University, Kampala, Kampala, Uganda; Infectious Diseases Institute, Makerere University, Kampala, Kampala, Uganda; Infectious Diseases Institute, Kampala, Kampala, Uganda; Infectious Diseases Institute, Kampala, Kampala, Uganda; Infectious Diseases Institute (IDI), Kampala City, Kampala, Uganda; Infectious Diseases Institute, Makerere University, Kampala, Kampala, Uganda; Infectious Diseases Institute, Makerere University, Kampala, Kampala, Uganda; University of Minnesota, Minneapolis, Minnesota; University of Minnesota, Minneapolis, Minnesota; University of Minnesota, Minneapolis, Minnesota; Mbarara University of Science and Technology, Mbarara city, Mbarara, Uganda; Infectious Diseases Institute, Makerere University, Kampala, Kampala, Uganda; University of Minnesota, Minneapolis, Minnesota

## Abstract

**Background:**

Cryptococcal meningitis is a leading cause of mortality among people living with HIV, especially among those with advanced HIV disease in Sub-Saharan Africa. Cryptococcosis causes an estimated 19% of HIV-related deaths worldwide. Elevated intracranial pressure (ICP, ≥ 200 mmH_2_O) is a common complication of cryptococcal meningitis. The causes, phenotypes, and clinical implications of persistently elevated ICP following treatment for cryptococcal meningitis are not well understood, but control of ICP has a survival benefit.

**Figure:**
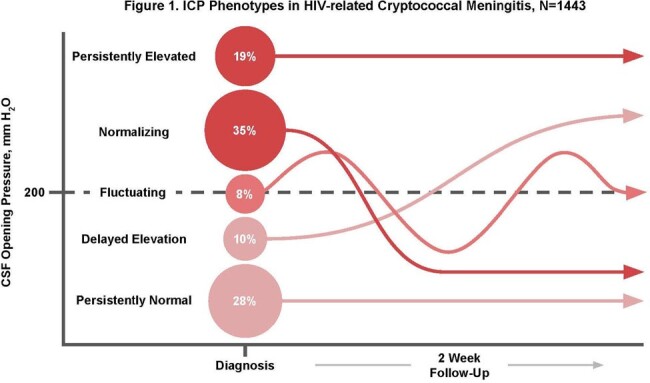

**Methods:**

We prospectively enrolled adults with HIV and cryptococcal meningitis in Kampala and Mbarara, Uganda from 2013 to 2023. Cerebrospinal fluid (CSF) opening pressure (OP) was recorded for each diagnostic and therapeutic lumbar puncture performed during hospitalization. We used the highest OP value in each follow-up time frame (days 2-4, 5-9, 10-16) to categorize participants into distinct ICP phenotypes. We assessed 2-week survival across ICP phenotypes.

**Figure:**
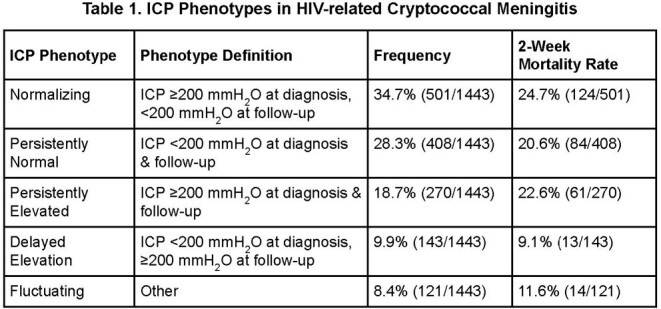

**Results:**

We enrolled 1443 participants with cryptococcal meningitis. Analysis of CSF OP revealed five distinct ICP phenotypes (Figure 1). First, 35% of participants had normalizing ICP defined as ICP ≥ 200 mmH_2_O at diagnosis, followed by ≥ 1 instance of ICP < 200 mmH_2_O with no subsequent increase. Second, 28% had persistently normal ICP with all OP values < 200 mmH_2_O. Third, 19% had persistently elevated ICP with all OP values ≥ 200 mmH_2_O. Fourth, 10% had delayed elevation in ICP defined as normal ICP at diagnosis, followed by ≥ 1 elevated ICP and no subsequent documented normalization. Fifth, 8% had fluctuating ICP that did not fit any other phenotype definition. Among these groups, 2-week mortality varied from 9% to 25% (Table 1). The normalizing, persistently elevated, and persistently normal phenotypes were associated with the poorest prognosis (log-rank test p < 0.001).

**Conclusion:**

This study highlights the heterogeneity of ICP phenotype patterns in cryptococcal meningitis. Further research is needed to investigate the underlying mechanisms and risk factors associated with these ICP phenotype groups and better anticipate clinical management needs.

**Disclosures:**

**All Authors**: No reported disclosures

